# In Vivo Imaging of Twist Drill Drainage for Subdural Hematoma: A Clinical Feasibility Study on Electrical Impedance Tomography for Measuring Intracranial Bleeding in Humans

**DOI:** 10.1371/journal.pone.0055020

**Published:** 2013-01-25

**Authors:** Meng Dai, Bing Li, Shijie Hu, Canhua Xu, Bin Yang, Jianbo Li, Feng Fu, Zhou Fei, Xiuzhen Dong

**Affiliations:** 1 Department of Biomedical Engineering, Fourth Military Medical University, Xi’an, China; 2 Neurosurgical Unit of Xijing Hospital, Fourth Military Medical University, Xi’an, China; University of California Berkeley, United States of America

## Abstract

Intracranial bleeding is one of the most severe medical emergencies in neurosurgery. Early detection or diagnosis would largely reduce the rate of disability and mortality, and improve the prognosis of the patients. Electrical Impedance Tomography (EIT) can non-invasively image the internal resistivity distribution within a human body using a ring of external electrodes, and is thus a promising technique to promptly detect the occurrence of intracranial bleedings because blood differs from other brain tissues in resistivity. However, so far there is no experimental study that has determined whether the intracranial resistivity changes in humans could be repeatedly detected and imaged by EIT. Hence, we for the first time attempt to clinically validate this by in vivo imaging the influx and efflux of irrigating fluid (5% dextrose in water, D5W) during the twist-drill drainage operation for the patients with subdural hematoma (SDH). In this study, six patients (four male, two female) with subacute or chronic SDH received the surgical operation in order to evacuate the hematoma around subdural region, and EIT measurements were performed simultaneously on each patient’s head. The results showed that the resistivity significantly increased on the corresponding position of EIT images during the influx of D5W and gradually decreased back to baseline during the efflux. In the quantitative analysis, the average resistivity values demonstrated the similar results and had highly linear correlation (R^2^ = 0.93±0.06) with the injected D5W volumes, as well as the area of the resistivity gain(R^2^ = 0.94±0.05). In conclusion, it was clinically validated that intracranial resistivity changes in humans were detectable and quantifiable by the EIT method. After further technical improvements, EIT has the great potential of being a routine neuroimaging tool for early detection of intracranial bleedings.

## Introduction

Intracranial bleeding (hemorrhage) occurs when a blood vessel within the skull is ruptured or leaks, giving rise to the pathological accumulation of blood within the cranial vault. It may be spontaneous, precipitated by an underlying vascular malformation, induced by trauma, or related to therapeutic anticoagulation, and the resulting increases in intracranial pressure (ICP) may crush delicate brain tissue, limit its blood supply, or even cause potentially fatal brain herniation [1]. Intracranial bleeding is generally considered as one of the most life-threatening conditions in neurosurgery. For example, subdural hematoma (SDH, a subtype of intracranial bleeding) has an incidence of 1–2 per 100,000 people per year and a mortality rate of around 60%–80% in acute cases [2], [3]. Corticosteroids, diuretics, or anticonvulsants are often clinically used as medications. In severe cases, surgical operation is performed to alleviate swelling and to prevent bleeding [1]. The goals of critical care are to assess the proximate cause, minimize the risks of hemorrhage expansion through blood pressure control and correction of coagulopathy, and obliterate vascular lesions with a high risk of acute re-bleeding. As these treatments become available, early diagnosis of intracranial bleeding is crucial for reducing the morbidity and the mortality [4].

Unfortunately, the immediate detection and diagnosis of intracranial bleedings by the regular methods appear challenging. In acute cases, intracranial bleedings are increasingly severe with larger hematomas and post-operative patients may experience re-bleeding episodes after the initial onset; although chronic intracranial bleedings have better prognosis if properly managed, they may be ignored and remain undiscovered until the presentation of symptoms, which contributes to poor outcomes [5]. Under these circumstances, clinical staffs may lose the chance of stopping episodes of bleeding before they cause significant damage. At present, CT scanning is usually the first evaluation done in patients with suspected intracranial bleedings [6]. However, although CT scanning shows hematomas many hours after bleeding onset, it cannot readily detect secondary bleeding during the acute time window, wherein patients may be treated with surgery or medical intervention [7]. CT scanning is not also practical for continuously monitoring patients with chronic intracranial bleedings at the bedside. Therefore, developing a non-invasive and radiation-free modality capable of promptly detecting the onset of intracranial bleedings and continuously monitoring its development is highly desirable.

Electrical impedance tomography (EIT) is a relatively new medical imaging technique that seeks to determine internal resistivity distributions within the body from current stimulation and electrical measurement at electrodes on the surface of an area of interest [8]. Several sequences of insensible current approximately 1 mA at high frequency (typically 50 kHz) are injected into the object and the corresponding boundary potentials are measured by a predefined set of electrodes; a cross-sectional EIT image is then calculated from these measurements; if the changes in measurements at two different moments are used to reconstruct images, it is referred to as “difference EIT”, which produces a difference image and reflects the relative changes in resistivity; the difference EIT has the ability to reduce the effect of many of the sources of error, and is thus applicable for a wide variety of problems. Considering its noninvasiveness and the absence of ionizing radiation, EIT has been proposed primarily as a monitoring and assessment tool for medical applications such as in detecting cancerous breast tissue [9], assessing gastrointestinal conditions or abdominal bleeding [10], and imaging of the ventilation and perfusion distribution in the thorax [11].

Furthermore, EIT may also be applied to imaging brain conditions, where changes of cortical impedance measured on the scalp are related to changes in local brain impedance. One of major applications in brain EIT is to image epilepsy and normal brain activity, which are systematically studied by the group at University College London (UCL). They first demonstrated that EIT with the UCLH Mark system could produce reproducible EIT images of epileptic seizures, functional activity and the phenomenon of spreading depression in anaesthetized experimental animals with a ring of electrodes on exposed brain [12]–[13]. With the development of brain modeling and image reconstruction [14]–[16], they also performed first clinical studies in humans in epileptic seizures and blood flow related changes over seconds in normal cortical evoked activity; a more controlled baseline measurement and less movement artifacts were reported as essential for clinical studies of brain EIT in the future [17]–[18].

As for intracranial bleedings, the principle by which EIT visualizes hematomas rest on the difference in the resistivity between blood and other brain tissues [19]. Through continuously monitoring the patients, EIT can provide a sequence of images of intracranial relative resistivity distribution which may indicate the information on hematomas. Murphy first attempted to detect intraventricular hemorrhage using the EIT method in one neonatal patient. However, these data were significantly affected by artifacts [20]. Shi established a multi-layered phantom to mimic the human head and imaged blood-like resistivity contrasts on the phantom using EIT [21]. In a recent study, Dai successfully detected subarachnoid hemorrhage in neonatal piglets with EIT and attempted to correlate the magnitude of resistivity changes with blood volumes [22]. Xu conducted a similar study on intracerebral hemorrhage as well [6]. Although these studies favorably showed that EIT was able to image intracranial bleedings on animal model, the further human research on whether intracranial resistivity changes could be repeatedly detected and imaged by EIT has not been reported in the literatures and the possibility of EIT imaging intracranial bleedings in humans needs further investigation.

Accordingly, our objective of the paper was to clinically validate this feasibility of EIT in humans by in vivo imaging the intracranial resistivity changes during twist-drill drainage operation for the patients with SDH. Since the irrigating fluid (5% dextrose in water, D5W) was injected and then drained out for cleaning the hematoma space, we could simultaneously perform difference EIT to observe the corresponding intracranial resistivity changes caused by D5W. Besides, we also performed relevant quantitative analysis by correlating the magnitude and the area of resistivity changes on EIT images with the volumes of D5W within skull. The reason we chose the SDH patients who needed twist-drill drainage treatment as the measured subjects are that (1) SDH is a typical intracranial bleeding; the observed phenomena on EIT for SDH may indicate the similar results for other intracranial bleeding. (2) Twist-drill drainage, unlike standard craniotomy which removes a large section of the skull, just makes a hole on skull and largely preserves the integrity of the head; the EIT results based on the model are relatively close to ones when performing non-invasively EIT measurements in real case.

## Materials and Methods

### Ethics Statement

The current study was approved by the Fourth Military Medical University Ethics Committee on Human Research and informed written consent was obtained from those SDH patients’ nearest relatives. The whole procedures were conducted according to the Principles of Helsinki.

### Patients

Six patients (four males and two females) participated in this study and their SDH resulted from physical trauma caused by accidental falls or traffic accidents. The vital signs of the patient group, including heart rate (HR, beats per minute), respiratory rate (RR, breaths per minute), blood pressure (BP, mmHg), as well as their Glasgow Coma Scale (GCS) scores and so on are given in Table 1.

**Table 1:** Characteristics of the SDH patients in this study.

Patient	Gender	Age	HR	RR	BP	GCS	CT location
A	Male	40	90	18	125/80	12	Rt frontal lobe
B	Female	57	80	17	106/60	13	Rt frontal lobe
C	Female	31	60	10	137/90	12	Rt frontal andtemporal lobe
D	Male	64	68	13	117/80	13	Lt frontal andtemporal lobe
E	Male	60	61	17	136/83	14	Lt frontal lobe
F	Male	68	85	21	125/70	11	Lt temporal lobe

In general, these patients developed sub-acute or chronic SDH because they did not have significant symptoms and not seek immediate treatment when the bleeding occurred. The occurrence of gradually increasing headaches, confusion, vomiting, or nausea several days later compelled their transfer to the ICU in the university hospital and twist drill drainage was the treatment of choice.

### Surgical Intervention

Although spontaneous resolution and successful non-surgical treatment have been reported [23]–[26], surgery is generally considered the best therapy for SDH [27]–[29]. The aim of surgical treatment is the decompression and removal of the fibrinolytic substances from the area. Among the available surgical treatments, evacuation of the SDH via twist drill craniotomy and closed-system drainage has been shown superior to the other methods [1].

In this study, all the patients were treated in the operating room with monitoring of their physiologic parameters (IntelliVue MP60, Philips Healthcare, Eindhoven, the Netherlands). After preparation of the operative field, general anesthesia, disinfection, and establishment of EIT electrodes, an incision approximately 3 cm in radius was made on the skin above or behind the top of the ear near the parietal eminence. Twist drill craniotomy was performed using a 3 mm hand-driven drill (Salxman Twist Drill Kit I, Elekta Corp., Stockholm, Sweden) and the diploe of the skull was cauterized with bipolar cautery (SYSTEM 2450™, ConMed Corp., New York, USA) to stop bleeding. The dura was perforated with a 16G cannula (BD Venflon™ IV cannula with PTFE radiopaque and injection valve, Franklin Lakes, USA) in the subdural space with great care. The hematoma was then discharged into a scaled tube inside the cannula. Considering rapid decompression is associated with intracerebral hematoma formation, overly brisk drainage was avoided. Afterwards, D5W was introduced and then drain out to help dislodge the residual clots. The procedure of irrigation and drainage was repeated until the return fluid was clear, and EIT data were measured at the same time. Finally, the distal end of the cannula was connected to a closed drainage system and the skin was closed with a single suture.

### Data Acquisition

**Figure 1:**
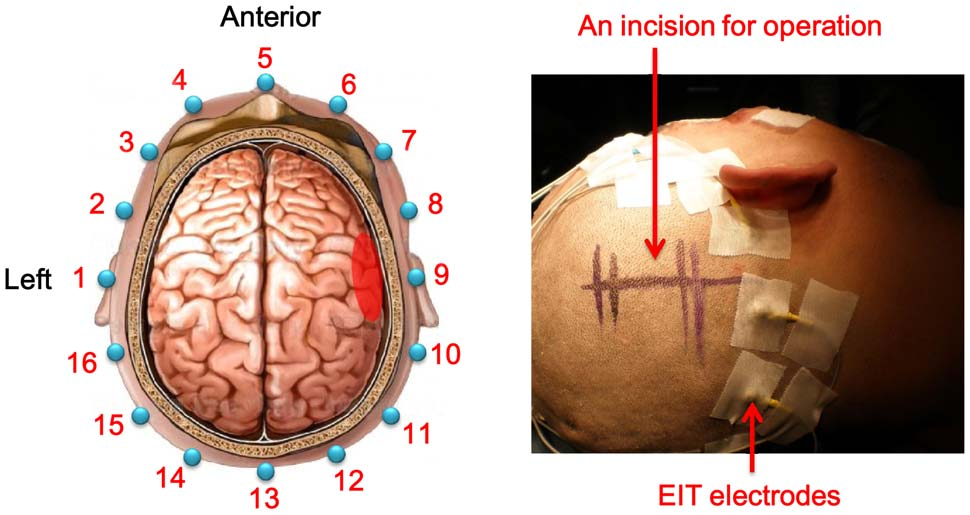
Establishment of brain EIT electrodes. The left part showed that 16 electrodes were equally spaced around the scalp and in the plane where hematoma existed. The right part showed the scene of operation where an incision was marked before cutting (the patient has given written informed consent, as outlined in the PLOS consent form, to publication of his photography).

EIT data were measured in real time using a brain-oriented EIT monitoring system developed by our group [30]. The working frequency of the system ranges from 1 kHz to 190 kHz, with the measuring accuracy at ±0.01% and the CMRR over 80 dB. Before the surgical operations, sixteen copper cup electrodes were rigorously sterilized and placed with the conductive gel (Ten20 conductive paste, Weaver and Company, Aurora, USA) on the circumference of the head cross-section where hematomas existed, below the operative incision (Figure 1). During the operations, the EIT system applied a safe alternating current (1 mA_p–p_, 50 kHz) into head using a pair of electrodes and the resulting surface potentials were measured between the remaining electrode pairs. Each data frame comprised 192 independent potentials over 1 sec. As a cycle of irrigation and drainage procedure often included a reference phase without influx, an influx phase and an efflux phase, EIT measurements were perform over those phases to observe corresponding resistivity changes on images. Since the first phase did not require any intervention, the EIT baseline measurements were carried out and the data were utilized as the reference frame for difference EIT. During an influx phase, D5W was then injected at a rate of 5 ml per minute and the simultaneous changes in resistivity were observed on the EIT images. When completing the injection of 20 ml D5W, we began to drain out the irrigating fluid at the same rate and continued performing difference EIT imaging. As the whole surgical operation included the 4–5 cycles of irrigation and drainage, 4–5 sequences of EIT images were obtained for each patient.

### Image Reconstruction

**Figure 2:**
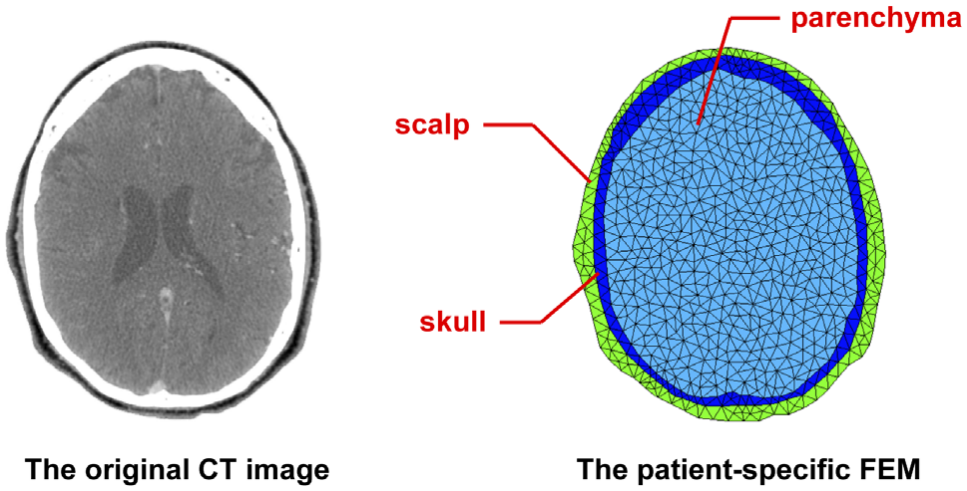
Establishment of a patient-specific FEM. The FEM was obtained by segmenting the original CT image into three parts (scalp, skull, parenchyma) and then discretizing them into those triangle elements. Each part was set to different resistivity as a prior information for the forward calculation. Considering the tradeoff between accuracy and computational cost, the number of elements was often set to 800–1000.

In order to improve the quality of EIT images, a patient-specific finite element model (FEM) for image reconstruction was obtained as follows. First, a single CT image of the patient’s head at the electrode plane was segmented into three parts: scalp, skull and parenchyma. Second, these three regions were discretized into triangle elements (totally 800–1000) and then respectively set to different resistivity (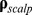 = 2.27 Ωm [16]; 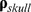 = 75.76 Ωm [31]; 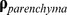 = 6.67 Ωm [16]) for the next calculation of the EIT forward problem (Figure 2).

In difference EIT, a vector of resistivity change 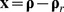 between the current resistivity 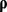 and the reference 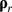 is reconstructed from measurements 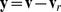 of the corresponding change in recorded voltage. For sufficiently small changes, the relationship between 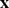 and 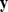 can be approximated by the linear relationship
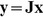
(1)


where 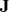 is the Jacobian or sensitivity matrix. In the forward problem, matrix 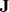 is calculated for each element of the FEM as 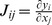. As the number of 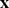 is larger than the number of 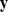, 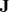 is not square and hence does not have an inverse. Instead, a linear reconstruction algorithm calculates an estimate of 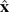 in the inverse problem. One general approach is to calculate 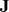
’s Moore-Penrose pseudo-inverse 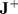 and a smoothed solution is achieved by



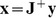
(2)In brain EIT, the entries of matrix 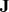 are relative small due to the resistive effect of the skull, representing low sensitivity within the head. We hence made the sensitivity more uniform using the weighted minimum norm method (WMNM) [32]. In WMNM, columns of the matrix 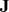 are equalized in terms of its power before pseudo-inversion and recovered by rescaling the pixels. The final form of WMNM is displayed in equation (3):
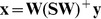
(3)where the weighting matrix 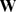 is a diagonal matrix whose entries are defined as 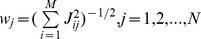. 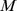 is the number of measurement, 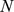 the number of elements.

### Image Analysis

As D5W was injected into the hematoma space within the skull, the corresponding resistivity changes might be shown in specific regions on the EIT images. In order to quantify the intracranial resistivity changes and to reduce influence caused by artifacts in the non-fluid regions, the average resistivity values (ARV) of the fluid region on the EIT images were calculated as follows [33]:
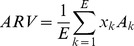
(4)where 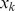 represents the reconstructed relative resistivity value on the kth fluid element, 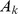 is the area and 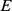 represents the total number of fluid elements. The identification of the fluid elements was determined through the reconstructed relative resistivity values using a predefined threshold:
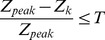
(5)where 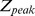 is the peak value of all elements, T the threshold parameter, which was set to 20% as previously suggested [33]. Furthermore, we defined the size of region of interest (sROI) to evaluate the area variation of the D5W fluid region on EIT images as follows,
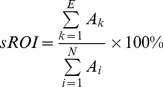
(6)where 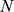 represents the total element number of the FEMs.

In statistical analysis, the Wilcoxon matched-pair method was applied to test the difference in ARV and sROI, respectively. All differences with P<0.05 were considered significant. Additionally, a linear regression analysis using the least square method (MATLAB 2009a, The MathWorks, Natick, USA) was applied to correlate the volumes of injected D5W against the ARV and the sROI. The Pearson’s correlation analysis was used to detect any correlation between these parameters.

### Absolute Resistivity Measurements

In order to compare the EIT results with the resistivity difference of the measured objects, we also conducted the resistivity measurements at 50 kHz (the frequency of EIT signals) of the living brain tissues and D5W using the two-terminal method via Agilent 4294A Impedance Analyzer (Agilent Technologies, Santa Clara, USA), which was installed near the neurosurgical operation room of the university hospital. The normal brain tissues were carefully derived from the excised brain tumor tissues and measured ex vivo within 10 minutes. Before the measurements, a measuring cell with parallel-plate capacitors was constructed for the purposes of fitting the cell to the analyzer’s test terminals. Two parameters of the measuring cell were routinely corrected to reduce the effect of electrode impedance, geometric error and stray capacitance: Cell constant (C0 = 0.097pF) and stray capacitance (C1 = 3.13pF) were determined by calibration with KCl standard solution at 25°C. The results were given in Table 2.

**Table 2:** The average values of the resistivity measured at 50 kHz for D5W and living brain tissues.

	D5W	Grey Matter	White Matter	Blood
Resistivity (Ωm)	129.83±14.77	6.67±2.53	7.17±1.45	1.64±1.05

### Calibration of the EIT System

**Figure 3:**
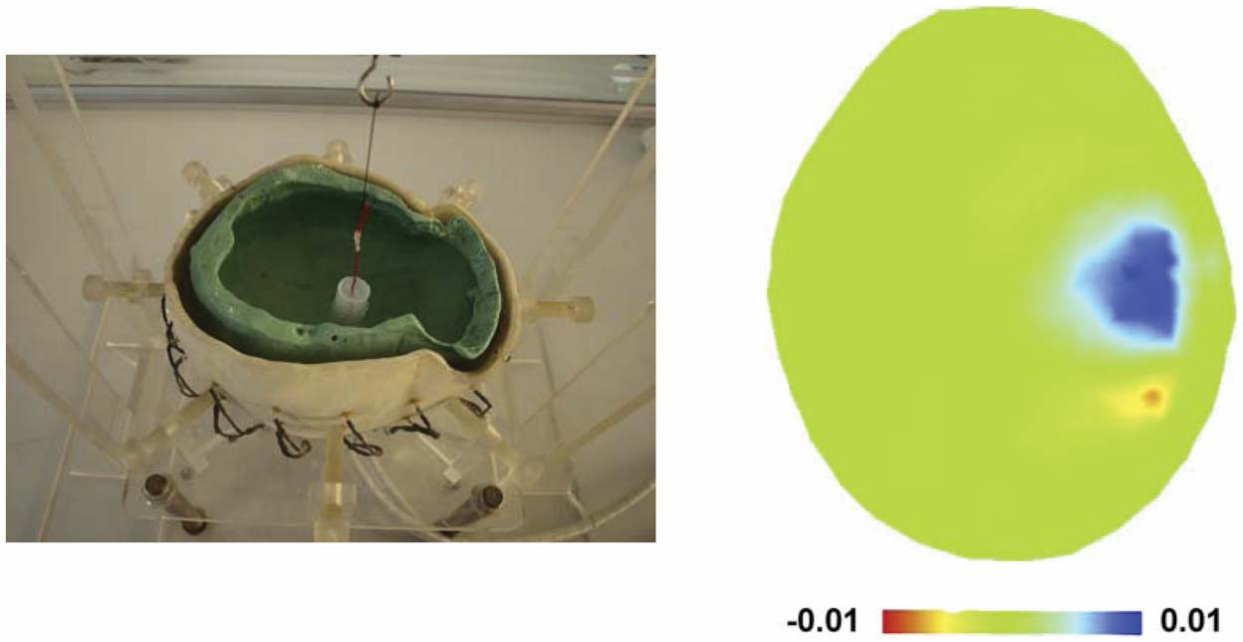
Calibration of the EIT system. The left showed the realistic human skull model made by plaster within a saline-filled tank; an agar cylinder was utilized to make perturbation. The right was the resulting EIT image with the agar cylinder of 20-fold increased resistivity and of 20 mm diameter.

**Figure 4:**
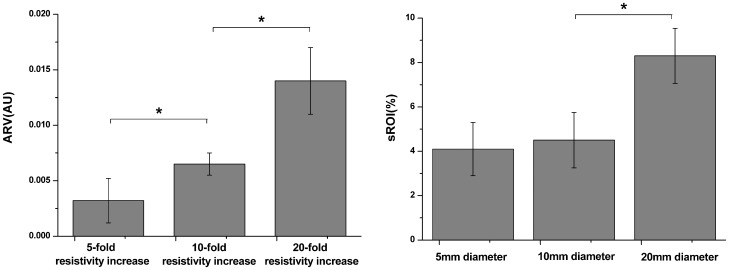
Comparison of the ARV for agar cylinders with different increased resistivity and comparison of the sROI for agar cylinders with different diameters. The left part demonstrated that the magnitude of ARV significantly increased with the resistivity of agar (* P<0.05); the right part also showed the sROI increased with the diameter of agar, but the sROI difference between the agar cylinders of 5 mm diameter and the ones of 10 mm diameter was insignificant.

As the EIT images were reconstructed using a new linear algorithm optimized for the head, the EIT system and algorithm were also calibrated by imaging objects within a 0.2% saline-filled tank which also contained a plaster model similar to a realistic human skull in terms of shape and resistivity. The electrodes were in identical position to those used on the patients. The agar cylinders with different resistivity increased (by 5, 10 and 20 times) and with different diameters (5, 10 and 20 mm) were served as imaging objects. In particular, we chose the objects of a 20-fold increased resistivity with respect to the dramatic resistivity difference between D5W and brain parenchyma as shown in Table 2. When the agar cylinder had 20-fold increased resistivity and 20 mm diameter, the reconstructed images localized it to within 9% of the image diameter of its true position in the tank. The full width at half maximum was 23% of the image diameter (Figure 3). As the resistivity of the agar cylinder of a constant diameter (20 mm) stepwise increased by different times, the magnitude of ARV in arbitrary unit (AU) accordingly grew up (P<0.05); when the resistivity of the agar cylinder was 20 times higher than that of saline, the magnitude of ARV reached up to 0.014±0.005(Mean ± SD). The sROI also tended to increase with the diameter of agar cylinders which had the same resistivity increase (20 fold), but the sROI difference between the agar cylinders of 5 mm diameter and the ones of 10 mm diameter appeared insignificant (P>0.05) (Figure 4).

## Results

In this study, all the surgical operations were successfully completed without any complications. After the operation, the patients were transferred to the ICU for further treatments.

**Figure 5:**
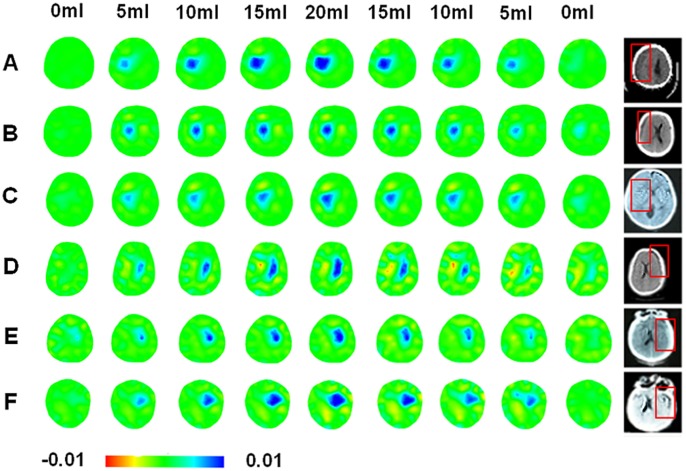
The EIT results of those SDH patients with twist drill drainage. The top texts indicated the intracranial D5W volumes. The first left column of EIT images did not show notable resistivity changes when no D5W injected, and served as the control groups. The most right column showed the original CT images for each patient and the positions of hematoma were indicated by those red boxes.

During baseline measurement, the relative resistivity changes remained stable and low (0.00020±0.00013 AU).The corresponding EIT images for all the patients during the irrigation and drainage of their hematoma are shown in Figure 5. For each patient, the first image on the left column showed a few changes in intensity when no D5W was injected, indicating that the relative resistivity changes due to normal physiological activities may not be great enough to be observed. As D5W was increasingly injected, the blue region gradually darkened and expanded, suggesting a gain in relative resistivity. The inverse course was also observed when draining out D5W. This phenomenon was repetitive in those six patients who underwent twist drill drainage.

**Figure 6:**
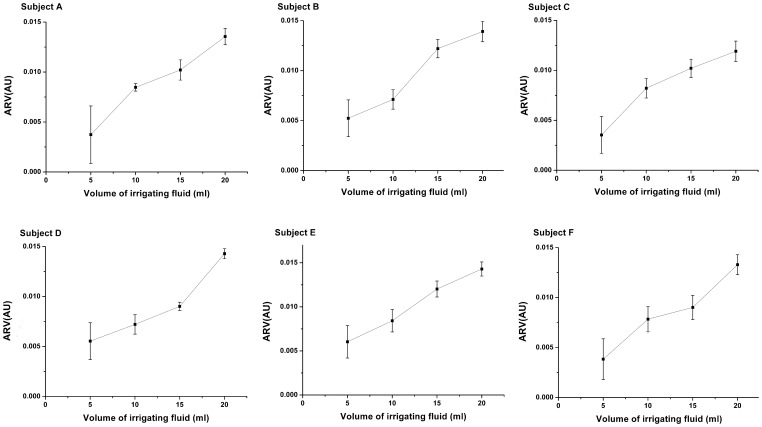
Plots of ARV against the volume of D5W. All the data during several drainage cycles were analyzed for each patient.

**Figure 7:**
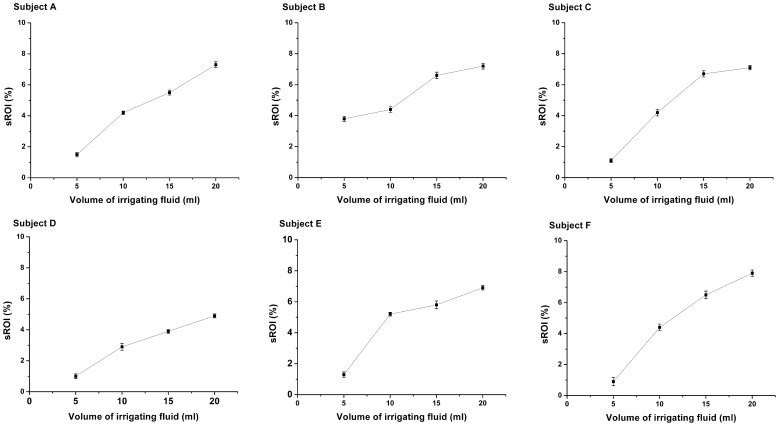
Plots of sROI against the volume of D5W. All the data during several irrigation and drainage cycles were analyzed for each patient.

**Figure 8:**
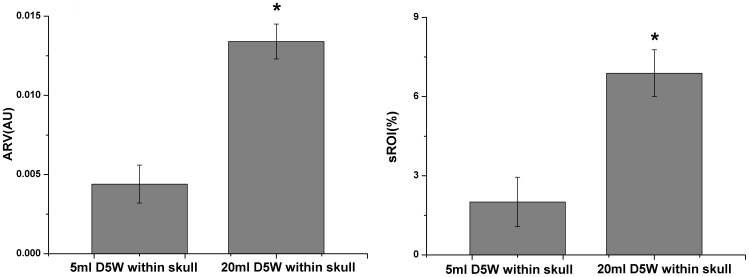
Comparison results in ARV and sROI. The ARV and sROI at 20 ml D5W within skull were both sigficantly (* P<0.05) larger than the ones at 5ml D5W.

The ARVs against the volumes of D5W from 5 ml to 20 ml were also analyzed for each patient in Figure 6. It was shown that the ARV varied from 0.001 to 0.015 and monotonically increased with the volumes of D5W, suggesting a possible mapping relationship between the ARV and the fluid volumes. The similar results were obtained by analyzing the sROI of the each patient for all drainage cycles (Figure 7). As the D5W was continuously injected, the sROI rose steadily in the range from 1%–8%. The results in Figure 8 also showed that the difference in the ARVs between the D5W volumes at 5 ml and at 20 ml was statistically significant, as well as the one in the sROI.

In addition, the correlation analysis showed that increase in the D5W volumes from 5 ml to 20 ml gave rise to a linear increase in ARV and sROI (P<0.05) (Table 3). Although the regression coefficients were different over those patients, the high determination coefficients demonstrated that the D5W volumes had the significant linear correlation with ARV (0.93±0.06) and with sROI (0.94±0.05).

**Table 3:** The results of linear correlation analysis of ARV and sROI against the volumes of D5W (* P<0.05).

	ARV		sROI	
Patient	Regression coefficient	Determination coefficient	Regression coefficient	Determination coefficient
A	6.82×10^−4^*	0.97	0.37*	0.98
B	6.52×10^−4^*	0.95	0.25*	0.94
C	5.82×10^−4^*	0.94	0.41*	0.92
D	5.48×10^−4^*	0.83	0.25*	0.97
E	5.50×10^−4^*	0.99	0.35*	0.85
F	6.22×10^−4^*	0.91	0.46*	0.96

Moreover, the absolute resistivity measured at 50 kHz demonstrated that the resistivity of D5W was around 20 times higher than the one of other brain tissues, and the calibration results also showed that the change magnitudes of EIT images induced by an agar cylinder with 20-fold increased resistivity in a realistic tank were close to that of EIT images in humans. Therefore, these facts confirmed that D5W was the primary cause of intracranial gain in resistivity and the magnitudes of EIT images were physiologically reasonable.

## Discussion

In this study, we for the first time provided the clinical evidence for the feasibility of EIT repeatedly detecting and imaging the intracranial resistivity changes in humans. This was achieved by in vivo imaging of the irrigating fluid within skull during the twist drill drainage for the SDH patients using EIT. With the stepwise increase in the injected D5W, a significant increase in resistivity was observed on the corresponding position of EIT images for each patient. The analysis of ARV and sROI were in concurrence with the results in EIT images and revealed that the magnitude and the area of relative changes in resistivity on EIT images were linearly correlated with the volumes of the irrigating fluid. This suggested that the intracranial resistivity changes be quantified by the indices extracted from the EIT images. As for intracranial bleedings, it may provide clinicians the chance of estimating the location and amount of hematomas within skull. In short, since the intracranial resistivity changes caused by D5W could be repeatedly imaged by the EIT method, there would be a great possibility of EIT detecting and quantifying the intracranial resistivity changes caused by hematomas as well.

Meanwhile, it must be realized that the detection of intracranial bleedings by EIT may still be challenging due to the existence of the skull [34]. The signals of intracranial resistivity changes may be attenuated by the resisting effect of the skull because it has an extremely higher resistivity as compared to brain parenchyma and shows resistivity inhomogeneity with different parts [31]. In this paper, D5W had the great difference in resistivity and thus yielded the notable and reproducible resistivity changes as indicated by EIT images. While the skull had an insignificant influence on imaging D5W, its negative effect on imaging intracranial hematomas in humans should be further investigated. One possible approach to the issue may lie on modeling the attenuation function of the skull for difference EIT signals. In this way, we are able to amplify this resistivity difference signals between blood and other brain tissues to the extent that they can be definitely detectable via EIT.

In addition to the impact of the skull, the EIT imaging is natural to be vulnerable to noise because it is typically an ill-posed and ill-conditioned inverse problem [35]. Although we simply averaged the measured data per ten seconds to reduce the noise level, some artifacts were also visible on the images (Patients D and F in Figure 3). Specifically in clinical settings of EIT application, patient movements or clinical manipulations will introduce severe noise interference corrupting the desired signals of resistivity changes. Therefore, besides optimizing the performance of EIT system for enhancing the anti-noise ability, we should established a synthetic signal processing method which is able to effectively reduce the measurement noise and extract the desired signals from the raw data measured.

Moreover, the accurate localization of anomalies is also an important issue to be addressed in EIT applications, especially in clinical monitoring. In a recent simulation study, Abascal showed that the use of the correct anisotropic FEM in image reconstruction, as opposed to an isotropic one, corrected an error of 24 mm in imaging a 10% resistivity increase located in the hippocampus, improved localization of resistivity changes deep in the brain due to epilepsy by 4–17 mm and led to a substantial improvement in image quality [16]. In our study, the FEM based on the realistic boundary extracted from each patient’s CT scan images was established for EIT reconstruction, but the anisotropy of brain tissues was ignored. This limitation may result in the errors for accurately localizing the resistivity changes. Therefore, in the future, we should also incorporate the anisotropy of brain tissues in humans into FEM to obtain an EIT image with more accurate localization.

Another limitation of this study is that the ROIs were simply defined based on reconstructed relative resistivity and the threshold was set to a constant value. The ROI-based analysis of EIT data aims to obtain information on regional brain lesions in the relevant area with respect to the underlying intracranial hematomas. Ideally, the ROIs selected for the analysis of brain-related phenomena should exhibit sufficient sensitivity to hematoma-induced changes in local electrical impedance. Thus, the ROIs should be defined individually depending on the subjects (e.g., adults or neonates, healthy or sick) and brain phenomena being analyzed, and a combination of the functional and arbitrary ROIs is recommended [36]. An optimum method of defining ROIs should objectively be chosen to quantify hematoma-related information.

In conclusion, it was clinically validated that intracranial resistivity changes in humans were repeatedly detectable and quantifiable by the EIT method. Further improvements in signal attenuation modeling, noise reduction, the anisotropic FEM, etc. would make EIT a reliable neuroimaging tool for early detection of intracranial bleedings in clinical practice.
